# Supplementary material for: Cost-effectiveness analysis of durvalumab as a maintenance treatment for patients with locally advanced, unresectable, stage Ⅲ nsclc in china
Source: PLoS One. 2022 Jun 24;17(6):e0270118. doi: 10.1371/journal.pone.0270118 (PMC9231800; doi:10.1371/journal.pone.0270118)
Supplement: S1 Fig — (PDF) [file pone.0270118.s001.pdf]

S1 Fig. KM data, active curve and outcome

The clinical efficacy data of both treatment groups were obtained from the patient-level data of the Asian ethnicity sub-population in the PACIFIC study which enrolled a total of 192 Asian patients, with 120 assigned to the durvalumab group and 72 to the BSC group.

According to Bayesian information criteria (BIC) and Akaike information criteria (AIC), visual inspection and statistical goodness-of-fit were conducted to determine the best fitting and extrapolation models for TTP, PFS and PPS curves in the PACIFIC study from six main survival functions including exponential, gen gamma, Gompertz, Weibull, log-logistic, and log-normal. The results showed that **generalized gamma** distribution represented the best goodness of fit to both TTP data and PFS data from patients in these two groups; and **Log-normal** distribution was the best fit distribution for PPS data from overall population.

1.TTP data

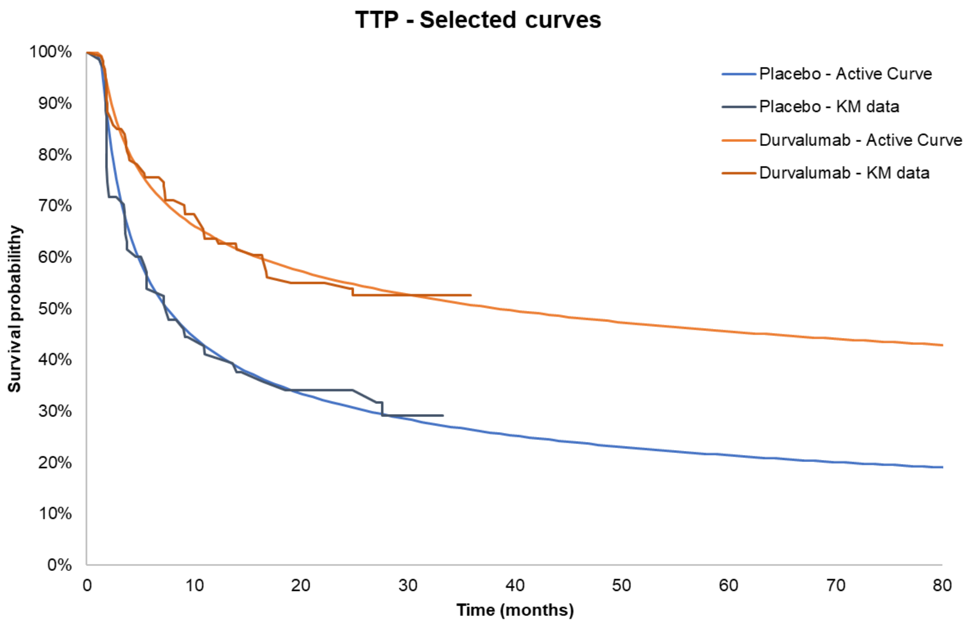

Original TTP curve

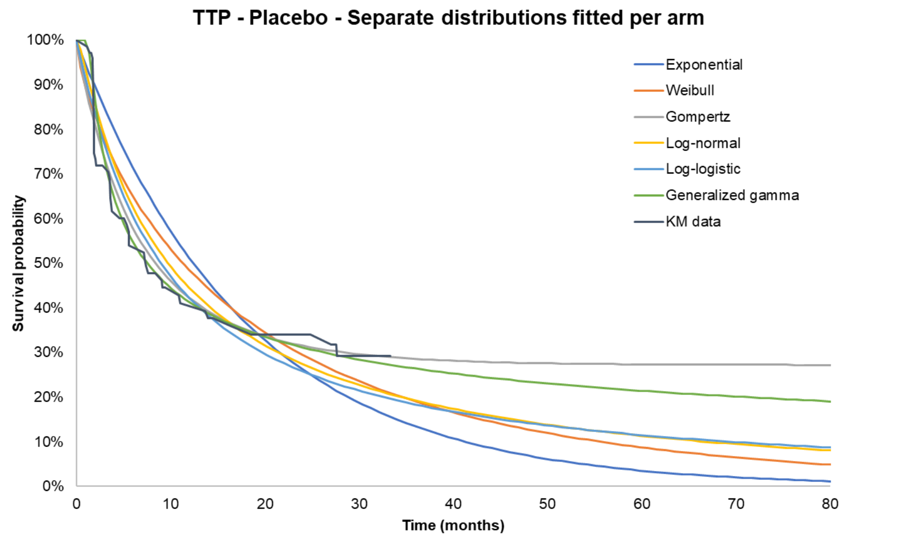

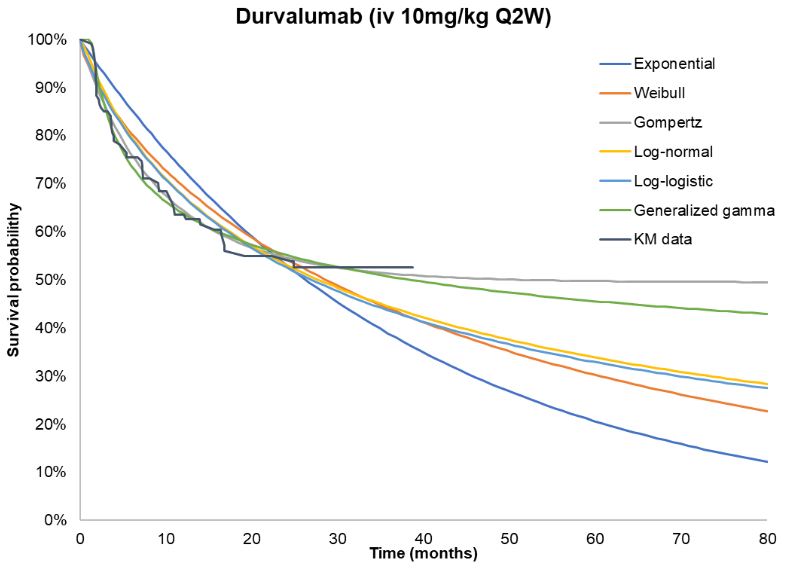

	AIC	BIC
**Generalized Gamma**	315	322
**Gompertz**	342	347
**Log-normal**	342	346
**Log-logistic**	346	350

	AIC	BIC
**Generalized Gamma**	446	454
**Gompertz**	467	472
**Log-normal**	469	475
**Log-logistic**	474	480

**Weibull**	355	360
**Exponential**	360	362

Placebo Best statistical fit: **Generalized Gamma**

**Weibull**	479	485
**Exponential**	484	487

Durvalumab Best statistical fit: **Generalized Gamma**

2.PFS data

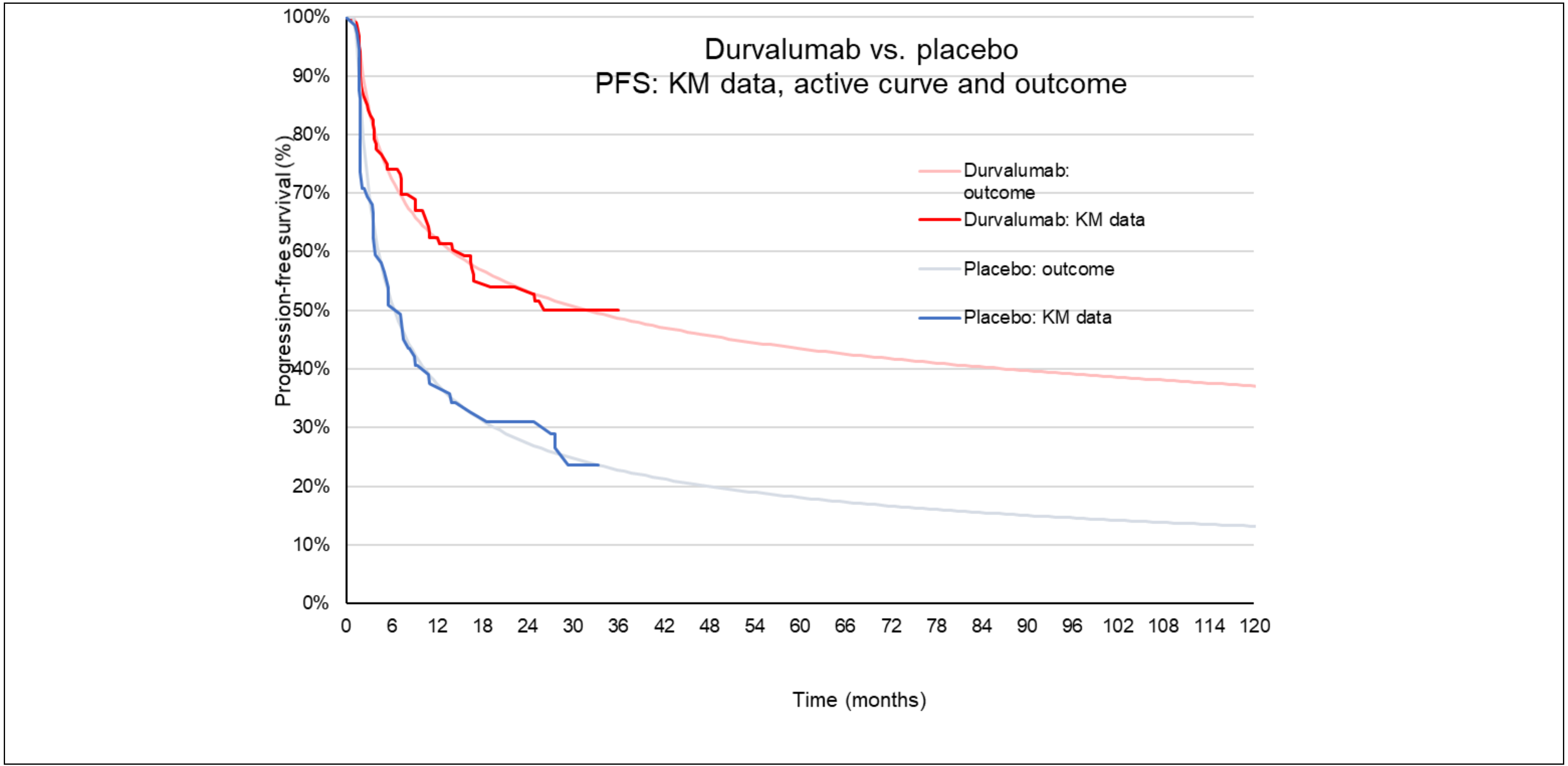
Durvalumab vs. placebo
PFS: KM data, active curve and outcome
Progression-free survival (%)
100%
90%
80%
70%
60%
50%
40%
30%
20%
10%
0%
0
6
12
18
24
30
36
42
48
54
60
66
72
78
84
90
96
102
108
114
120
Time (months)
Durvalumab: outcome
Durvalumab: KM data
Placebo: outcome
Placebo: KM data

Original PFS curve

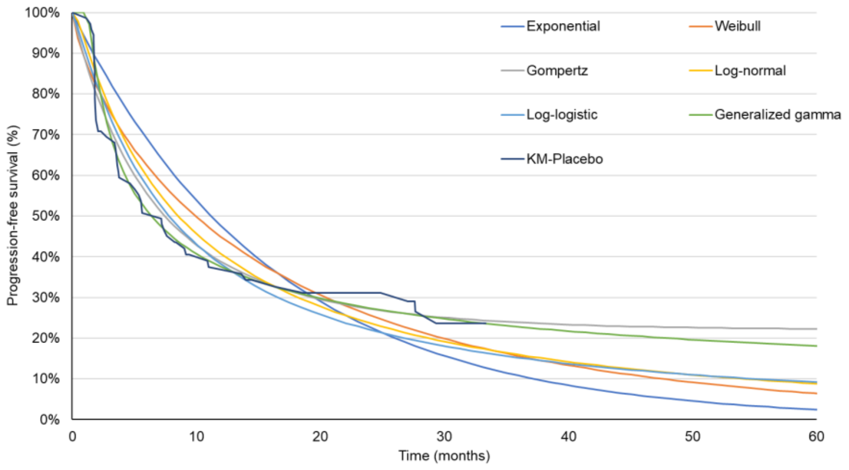

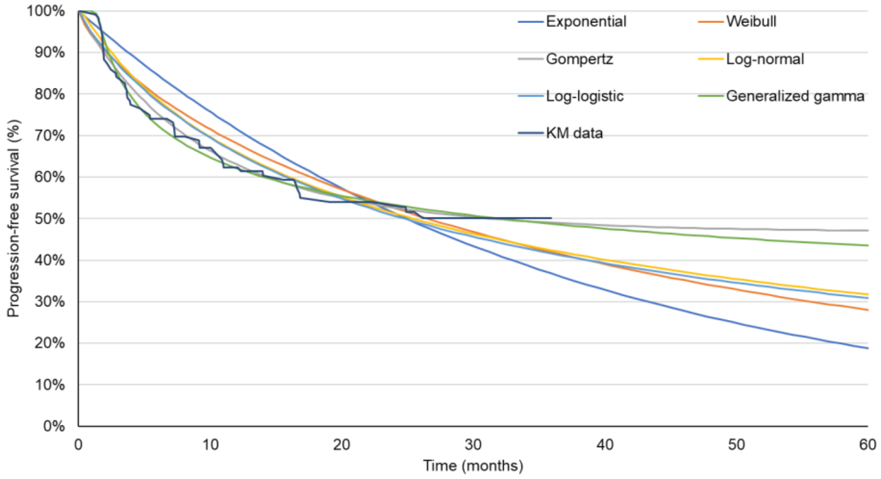

	AIC	BIC
Exponential	388	390
Generalized gamma	341	348
Gompertz	372	376
Log-logistic	373	377

	AIC	BIC
Exponential	506	509
Generalized gamma	466	474
Gompertz	489	494
Log-logistic	496	501

Log-normal	369	374
Weibull	384	388

Placebo Best statistical fit: **Generalized Gamma**

Log-normal	490	496
Weibull	501	507

Durvalumab Best statistical fit: **Generalized Gamma**

3.PPS data

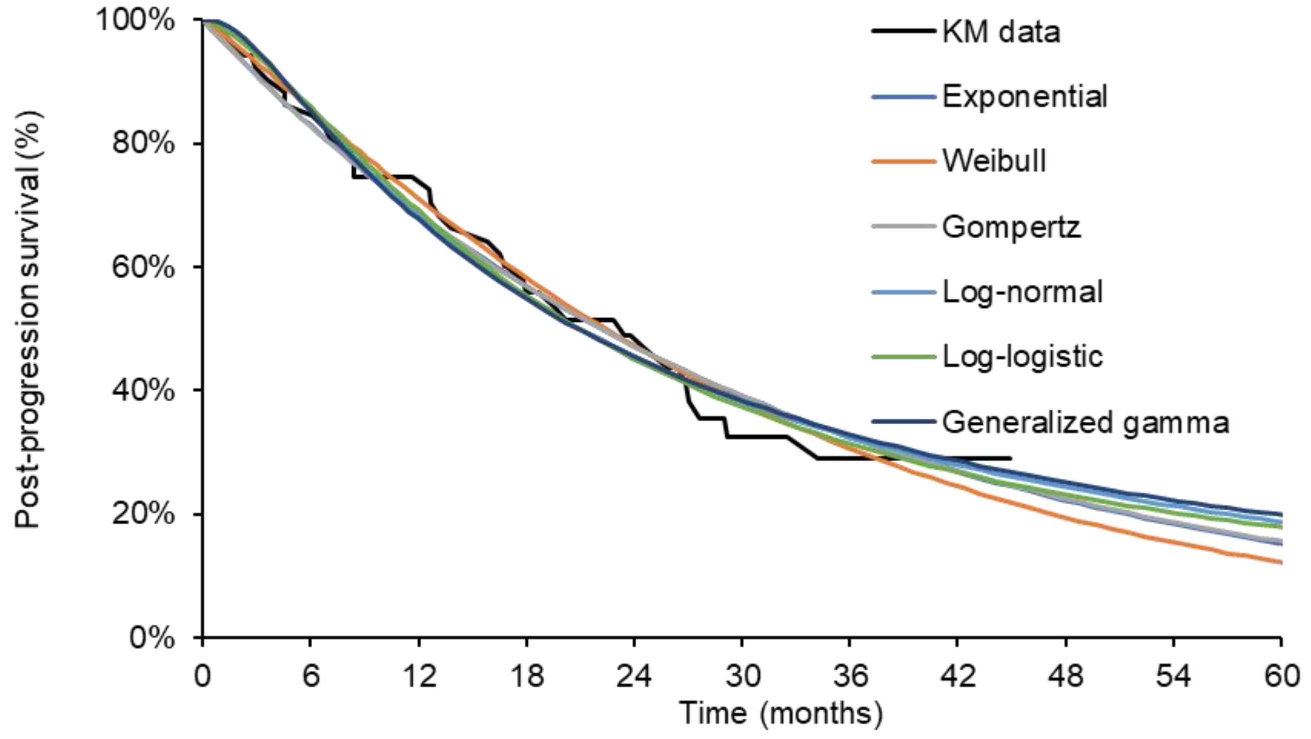

Original PPS curve

	AIC	BIC
Generalized Gamma	526.94	534.7
Log-normal	525.02	530.19
Log-logistic	526.37	531.54
Exponential	528.91	531.49
Weibull	529.77	534.94
Gompertz	530.9	536.07

Best statistical fit: **Log-normal**
