# Supplementary material for: Cost-effectiveness analysis of durvalumab as a maintenance treatment for patients with locally advanced, unresectable, stage Ⅲ nsclc in china
Source: PLoS One. 2022 Jun 24;17(6):e0270118. doi: 10.1371/journal.pone.0270118 (PMC9231800; doi:10.1371/journal.pone.0270118)
Supplement: S1 Table — (PDF) [file pone.0270118.s002.pdf]

S2 Table. The original clinical efficacy data from the PACIFIC study

1.Original TTP data

Placebo

time	survival	n.risk	n.event	std.err	lower 95% CI	upper 95% CI
0.000	1.000	0.000	0.000	0.000	1.000	0.998
1.150	0.986	72.000	1.000	0.014	0.905	0.998
1.446	0.972	71.000	1.000	0.019	0.893	0.993
1.544	0.972	70.000	0.000	0.019	0.893	0.993
1.708	0.958	69.000	1.000	0.024	0.876	0.986
1.741	0.944	68.000	1.000	0.027	0.858	0.979
1.774	0.888	67.000	4.000	0.037	0.788	0.942
1.807	0.874	63.000	1.000	0.039	0.771	0.932
1.840	0.803	62.000	5.000	0.047	0.690	0.878
1.873	0.775	57.000	2.000	0.050	0.659	0.856
1.906	0.747	55.000	2.000	0.052	0.629	0.832
2.004	0.733	53.000	1.000	0.053	0.614	0.820
2.070	0.719	52.000	1.000	0.053	0.599	0.808
2.333	0.719	51.000	0.000	0.053	0.599	0.808
2.760	0.719	50.000	0.000	0.053	0.599	0.808
3.417	0.704	49.000	1.000	0.054	0.583	0.796
3.515	0.689	48.000	1.000	0.055	0.567	0.783

3.614	0.645	47.000	3.000	0.057	0.522	0.745
3.713	0.631	44.000	1.000	0.058	0.507	0.732
3.778	0.616	43.000	1.000	0.058	0.492	0.718
4.600	0.601	42.000	1.000	0.059	0.477	0.705
4.632	0.601	41.000	0.000	0.059	0.477	0.705
5.027	0.601	40.000	0.000	0.059	0.477	0.705
5.355	0.586	39.000	1.000	0.059	0.461	0.691
5.552	0.570	38.000	1.000	0.060	0.446	0.677
5.585	0.555	37.000	1.000	0.060	0.430	0.663
5.618	0.540	36.000	1.000	0.060	0.415	0.649
7.162	0.524	35.000	1.000	0.060	0.400	0.634
7.228	0.509	34.000	1.000	0.061	0.385	0.620
7.392	0.493	33.000	1.000	0.061	0.370	0.605
7.622	0.478	32.000	1.000	0.061	0.356	0.590
8.246	0.478	31.000	0.000	0.061	0.356	0.590
8.345	0.478	30.000	0.000	0.061	0.356	0.590
9.035	0.461	29.000	1.000	0.061	0.340	0.575
9.166	0.445	28.000	1.000	0.061	0.324	0.559
9.429	0.445	27.000	0.000	0.061	0.324	0.559
10.908	0.428	26.000	1.000	0.061	0.308	0.543
11.006	0.411	25.000	1.000	0.061	0.292	0.526
13.635	0.394	24.000	1.000	0.061	0.276	0.509

13.996	0.377	23.000	1.000	0.060	0.260	0.492
14.489	0.377	22.000	0.000	0.060	0.260	0.492
16.361	0.359	21.000	1.000	0.060	0.244	0.475
18.595	0.341	20.000	1.000	0.060	0.227	0.457
22.012	0.341	19.000	0.000	0.060	0.227	0.457
22.341	0.341	18.000	0.000	0.060	0.227	0.457
24.641	0.341	17.000	0.000	0.060	0.227	0.457
24.871	0.341	16.000	0.000	0.060	0.227	0.457
27.072	0.318	15.000	1.000	0.060	0.206	0.436
27.565	0.318	14.000	0.000	0.060	0.206	0.436
27.598	0.318	13.000	0.000	0.060	0.206	0.436
27.630	0.291	12.000	1.000	0.060	0.180	0.412
29.306	0.291	9.000	0.000	0.060	0.180	0.412
30.357	0.291	8.000	0.000	0.060	0.180	0.412
30.423	0.291	7.000	0.000	0.060	0.180	0.412
30.620	0.291	6.000	0.000	0.060	0.180	0.412
32.920	0.291	5.000	0.000	0.060	0.180	0.412
33.117	0.291	4.000	0.000	0.060	0.180	0.412
33.150	0.291	3.000	0.000	0.060	0.180	0.412
33.314	0.291	2.000	0.000	0.060	0.180	0.412
35.877	0.291	1.000	0.000	0.060	0.180	0.412

Durvalumab

time	survival	n.risk	n.event	std.err	lower 95%CI	upper 95%CI
0.000	1.000	0.000	0.000	0.000	1.000	0.999
1.281	0.992	120.000	1.000	0.008	0.942	0.999
1.511	0.983	119.000	1.000	0.012	0.935	0.996
1.544	0.975	118.000	1.000	0.014	0.925	0.992
1.676	0.967	117.000	1.000	0.016	0.914	0.987
1.741	0.950	116.000	2.000	0.020	0.892	0.977
1.807	0.942	114.000	1.000	0.021	0.882	0.972
1.840	0.917	113.000	3.000	0.025	0.851	0.954
1.873	0.908	110.000	1.000	0.026	0.841	0.948
1.906	0.900	109.000	1.000	0.027	0.831	0.942
1.938	0.883	108.000	2.000	0.029	0.811	0.929
2.136	0.875	106.000	1.000	0.030	0.801	0.923
2.300	0.867	105.000	1.000	0.031	0.792	0.916
2.464	0.858	104.000	1.000	0.032	0.782	0.909
2.825	0.850	103.000	1.000	0.033	0.773	0.903
2.891	0.850	101.000	0.000	0.033	0.773	0.903
3.187	0.850	100.000	0.000	0.033	0.773	0.903
3.548	0.841	99.000	1.000	0.033	0.763	0.896
3.581	0.833	98.000	1.000	0.034	0.753	0.889
3.647	0.824	97.000	1.000	0.035	0.743	0.882

3.680	0.816	96.000	1.000	0.036	0.734	0.875
3.713	0.807	95.000	1.000	0.036	0.724	0.867
3.910	0.798	94.000	1.000	0.037	0.715	0.860
3.943	0.790	93.000	1.000	0.037	0.705	0.853
4.632	0.781	92.000	1.000	0.038	0.696	0.846
5.388	0.764	91.000	2.000	0.039	0.677	0.831
5.454	0.756	89.000	1.000	0.040	0.668	0.823
5.651	0.756	88.000	0.000	0.040	0.668	0.823
6.735	0.756	87.000	0.000	0.040	0.668	0.823
7.195	0.747	86.000	1.000	0.040	0.658	0.816
7.228	0.738	85.000	1.000	0.040	0.649	0.808
7.294	0.729	84.000	1.000	0.041	0.639	0.800
7.326	0.711	82.000	2.000	0.042	0.620	0.784
7.688	0.711	80.000	0.000	0.042	0.620	0.784
8.115	0.711	79.000	0.000	0.042	0.620	0.784
9.133	0.702	78.000	1.000	0.042	0.610	0.776
9.199	0.684	76.000	2.000	0.043	0.591	0.760
10.053	0.684	73.000	0.000	0.043	0.591	0.760
10.875	0.655	72.000	3.000	0.044	0.561	0.734
10.973	0.646	68.000	1.000	0.045	0.551	0.726
11.006	0.636	67.000	1.000	0.045	0.541	0.717
12.123	0.636	65.000	0.000	0.045	0.541	0.717

12.320	0.626	64.000	1.000	0.045	0.530	0.708
12.419	0.626	63.000	0.000	0.045	0.530	0.708
13.832	0.626	62.000	0.000	0.045	0.530	0.708
13.963	0.626	61.000	0.000	0.045	0.530	0.708
14.029	0.616	60.000	1.000	0.046	0.519	0.698
15.639	0.605	59.000	1.000	0.046	0.508	0.689
16.230	0.605	57.000	0.000	0.046	0.508	0.689
16.394	0.605	56.000	0.000	0.046	0.508	0.689
16.493	0.594	55.000	1.000	0.047	0.497	0.679
16.624	0.583	54.000	1.000	0.047	0.485	0.669
16.789	0.572	53.000	1.000	0.048	0.474	0.659
16.821	0.561	52.000	1.000	0.048	0.462	0.649
19.154	0.550	51.000	1.000	0.048	0.451	0.639
19.351	0.550	50.000	0.000	0.048	0.451	0.639
19.548	0.550	49.000	0.000	0.048	0.451	0.639
22.308	0.550	48.000	0.000	0.048	0.451	0.639
24.641	0.539	47.000	1.000	0.049	0.439	0.628
24.871	0.539	46.000	0.000	0.049	0.439	0.628
24.903	0.526	42.000	1.000	0.049	0.425	0.616
25.101	0.526	38.000	0.000	0.049	0.425	0.616
25.495	0.526	36.000	0.000	0.049	0.425	0.616
26.185	0.526	35.000	0.000	0.049	0.425	0.616

27.203	0.526	34.000	0.000	0.049	0.425	0.616
27.466	0.526	33.000	0.000	0.049	0.425	0.616
27.499	0.526	32.000	0.000	0.049	0.425	0.616
27.532	0.526	31.000	0.000	0.049	0.425	0.616
27.598	0.526	29.000	0.000	0.049	0.425	0.616
27.663	0.526	28.000	0.000	0.049	0.425	0.616
27.696	0.526	27.000	0.000	0.049	0.425	0.616
29.175	0.526	26.000	0.000	0.049	0.425	0.616
29.569	0.526	25.000	0.000	0.049	0.425	0.616
30.160	0.526	24.000	0.000	0.049	0.425	0.616
30.193	0.526	22.000	0.000	0.049	0.425	0.616
30.324	0.526	21.000	0.000	0.049	0.425	0.616
30.357	0.526	19.000	0.000	0.049	0.425	0.616
30.456	0.526	18.000	0.000	0.049	0.425	0.616
30.554	0.526	17.000	0.000	0.049	0.425	0.616
30.850	0.526	15.000	0.000	0.049	0.425	0.616
32.920	0.526	14.000	0.000	0.049	0.425	0.616
33.183	0.526	13.000	0.000	0.049	0.425	0.616
33.216	0.526	12.000	0.000	0.049	0.425	0.616
33.248	0.526	11.000	0.000	0.049	0.425	0.616
33.281	0.526	9.000	0.000	0.049	0.425	0.616
33.347	0.526	8.000	0.000	0.049	0.425	0.616

33.478	0.526	7.000	0.000	0.049	0.425	0.616
35.713	0.526	6.000	0.000	0.049	0.425	0.616
35.778	0.526	4.000	0.000	0.049	0.425	0.616
35.844	0.526	3.000	0.000	0.049	0.425	0.616
35.910	0.526	2.000	0.000	0.049	0.425	0.616
38.702	0.526	1.000	0.000	0.049	0.425	0.616

2.Original PFS data

Durvalumab

No. at risk	Time (months)	Survival
0.000	0.000	1.000
120.000	1.281	0.992
119.000	1.511	0.983
118.000	1.544	0.975
117.000	1.676	0.967
116.000	1.741	0.950
114.000	1.807	0.942
113.000	1.840	0.917
110.000	1.873	0.908
109.000	1.906	0.900
108.000	1.938	0.883
106.000	2.136	0.875
105.000	2.300	0.867
104.000	2.464	0.858
103.000	2.825	0.850
101.000	2.891	0.842
100.000	3.187	0.833
99.000	3.548	0.825

98.000	3.581	0.816
97.000	3.647	0.808
96.000	3.680	0.800
95.000	3.713	0.791
94.000	3.910	0.783
93.000	3.943	0.774
92.000	4.632	0.766
91.000	5.388	0.749
89.000	5.454	0.741
88.000	5.651	0.741
87.000	6.735	0.741
86.000	7.195	0.732
85.000	7.228	0.723
84.000	7.294	0.715
82.000	7.326	0.697
80.000	7.688	0.697
79.000	8.115	0.697
78.000	9.133	0.688
76.000	9.199	0.670
73.000	10.053	0.670
72.000	10.875	0.642
68.000	10.973	0.633

67.000	11.006	0.623
65.000	12.123	0.623
64.000	12.320	0.614
63.000	12.419	0.614
62.000	13.832	0.614
61.000	13.963	0.614
60.000	14.029	0.603
59.000	15.639	0.593
57.000	16.230	0.593
56.000	16.394	0.593
55.000	16.493	0.582
54.000	16.624	0.572
53.000	16.789	0.561
52.000	16.821	0.550
51.000	19.154	0.539
50.000	19.351	0.539
49.000	19.548	0.539
48.000	22.308	0.539
47.000	24.641	0.528
46.000	24.871	0.528
42.000	24.903	0.515
38.000	25.101	0.515

36.000	25.495	0.515
35.000	26.185	0.501
34.000	27.203	0.501
33.000	27.466	0.501
32.000	27.499	0.501
31.000	27.532	0.501
29.000	27.598	0.501
28.000	27.663	0.501
27.000	27.696	0.501
26.000	29.175	0.501
25.000	29.569	0.501
24.000	30.160	0.501
22.000	30.193	0.501
21.000	30.324	0.501
19.000	30.357	0.501
18.000	30.456	0.501
17.000	30.554	0.501
15.000	30.850	0.501
14.000	32.920	0.501
13.000	33.183	0.501
12.000	33.216	0.501
11.000	33.248	0.501

9.000	33.281	0.501
8.000	33.347	0.501
7.000	33.478	0.501
6.000	35.713	0.501
4.000	35.778	0.501
3.000	35.844	0.501
2.000	35.910	0.501
1.000	38.702	0.501

Placebo

Time (months)	No. at risk	Survival
0	0	1
1.1499	72	0.98611
1.44559	71	0.97222
1.54415	70	0.95833
1.70842	69	0.94444
1.74127	68	0.93056
1.77413	67	0.875
1.80698	63	0.86111
1.83984	62	0.79167
1.87269	57	0.76389
1.90554	55	0.73611
2.00411	53	0.72222

2.06982	52	0.70833
2.33265	51	0.70833
2.75975	50	0.69417
3.41684	49	0.68
3.5154	48	0.66583
3.61396	47	0.62333
3.71253	44	0.60917
3.77823	43	0.595
4.59959	42	0.58083
4.63244	41	0.58083
5.02669	40	0.56631
5.35524	39	0.55179
5.55236	38	0.53727
5.58522	37	0.52275
5.61807	36	0.50823
7.16222	35	0.49371
7.22793	34	0.47919
7.3922	33	0.46467
7.62218	32	0.45015
8.24641	31	0.43563
8.34497	30	0.43563
9.03491	29	0.4206

9.16632	28	0.40558
9.42916	27	0.40558
10.9076	26	0.38998
11.00616	25	0.37438
13.6345	24	0.35878
13.99589	23	0.34318
14.48871	22	0.34318
16.3614	21	0.32684
18.59548	20	0.3105
22.01232	19	0.3105
22.34086	18	0.3105
24.64066	17	0.3105
24.87064	16	0.3105
27.07187	15	0.2898
27.56468	14	0.2898
27.59754	13	0.2898
27.63039	12	0.26565
29.30595	9	0.23613
30.35729	8	0.23613
30.423	7	0.23613
30.62012	6	0.23613
32.91992	5	0.23613

33.11704	4	0.23613
33.1499	3	0.23613
33.31417	2	0.23613
35.8768	1	0.23613

3.Original PPS data

Time (months)	Suvival	N at risk
1.01848	0.98077	52
1.67556	0.96154	51
2.33265	0.94231	50
2.66119	0.94231	49
2.95688	0.92268	48
3.61396	0.90304	47
4.56674	0.88341	46
4.59959	0.86378	45
6.11088	0.84415	44
6.76797	0.82452	43
7.16222	0.80489	42
8.21355	0.78526	41
8.41068	0.76562	40
8.44353	0.74599	39
9.56057	0.74599	38
11.63039	0.74599	37
12.58316	0.72527	36
12.68172	0.70455	35
13.10883	0.68383	34
13.83162	0.66311	33

15.83573	0.64238	32
16.52567	0.62166	31
16.7885	0.60094	30
16.88706	0.60094	29
17.77413	0.57948	28
17.97125	0.55802	27
18.69405	0.55802	26
19.54825	0.53569	25
20.20534	0.51337	24
22.6037	0.51337	23
22.83368	0.51337	22
23.45791	0.48893	21
23.75359	0.48893	20
24.73922	0.46319	19
25.85626	0.43746	18
26.44764	0.43746	17
26.87474	0.41012	16
27.07187	0.38278	15
27.63039	0.35544	14
28.97741	0.35544	13
29.17454	0.32582	12
30.16016	0.32582	11

32.49281	0.32582	10
34.20123	0.28962	9
35.8768	0.28962	8
35.90965	0.28962	7
36.23819	0.28962	6
37.58522	0.28962	5
39.91786	0.28962	4
42.41478	0.28962	3
44.87885	0.28962	2
