# Supplementary material for: Cost-effectiveness analysis of durvalumab as a maintenance treatment for patients with locally advanced, unresectable, stage Ⅲ nsclc in china
Source: PLoS One. 2022 Jun 24;17(6):e0270118. doi: 10.1371/journal.pone.0270118 (PMC9231800; doi:10.1371/journal.pone.0270118)
Supplement: Attachment — Submitted filename: Authors response.docx [file pone.0270118.s003.docx]

**Response to Reviewers**

Dear Jianxin Xue and reviewers,

Thank you for your letter and the reviewers’ comments on our manuscript entitled “COST-EFFECTIVENESS ANALYSIS OF DURVALUMAB AS A MAINTENANCE TREATMENT FOR PATIENTS WITH LOCALLY ADVANCED, UNRESECTABLE, STAGE Ⅲ NSCLC IN CHINA”(manuscript number: PONE-D-21-27815). Those comments are very helpful for revising and improving our paper, as well as the important guiding significance to other research. We have studied the comments carefully and made corrections which we hope meet with approval. The main corrections are in the manuscript and the responds to the reviewers’ comments are as follows(the replies are highlighted in blue).

Replies to the reviewers’ comments:

**Reviewer #1:**

1. The study comments on the UK cost-effectiveness perspective of Witlox W et al stating that "Durvalumab was not cost-effective because the unmatured of survival data". I think it is worthy to cite the updated perspective after 4 years survival in the report: Dunlop, W., van Keep, M., Elroy, P. et al. Cost Effectiveness of Durvalumab in Unresectable Stage III NSCLC: 4-Year Survival Update and Model Validation from a UK Healthcare Perspective. PharmacoEconomics Open (2021). https://doi.org/10.1007/s41669-021-00301-7.

Response: **We have replaced the reference and updated the values in Table 4 as well as the descriptions in the text.**

2. China is a big market. It would be helpful to clarify the way of curation of drugs. Is it a free market with governmental licensing or a central curation authority able to negotiate price discounts, that would affect cost-effectiveness evaluations by the power of monopsony.

Response: **It is a central curation authority able to negotiate price discounts. The National Healthcare Security Administration, NHSA manages the price negotiation, known as the National Reimbursement Drug List (NRDL). Drugs that make it on this list are covered by national health insurance, available to nearly all Chinese citizens today.**

**Reviewer #2:**

1. The paper is solid from the statistical point of view, but the authors should explain in more detail the importance of comparing these two treatments beyond costs. Has Durvalumab following cCRT better results than BSC?

Response: **The life years of durvalumab group (7.39 years) were 2.6 years longer than those of BSC group (4.79 years); and more QALYs gained in durvalumab group (6.61 QALYs) than those in BSC group (4.24 QALYs).**

Kind regards.

Corresponding author: Jinyu Chen

E-mail address: [sycjy1015@163.com](mailto:sycjy1015@163.com)
